# Optical imaging of tumor vascularity associated with proliferation and glucose metabolism in early breast cancer: clinical application of total hemoglobin measurements in the breast

**DOI:** 10.1186/1471-2407-13-514

**Published:** 2013-10-31

**Authors:** Shigeto Ueda, Noriko Nakamiya, Kazuo Matsuura, Takashi Shigekawa, Hiroshi Sano, Eiko Hirokawa, Hiroko Shimada, Hiroaki Suzuki, Motoki Oda, Yutaka Yamashita, Osamu Kishino, Ichiei Kuji, Akihiko Osaki, Toshiaki Saeki

**Affiliations:** 1 Department of Breast Oncology, International Medical Center, Saitama Medical University, Hidaka City 350-1298, Saitama, Japan; 2 Department of Nuclear Medicine, International Medical Center, Saitama Medical University, Hidaka City 350-1298, Saitama, Japan; 3 Central US Service, International Medical Center, Saitama Medical University, Hidaka City 350-1298, Saitama, Japan; 4 Central Research Laboratory, Hamamatsu Photonics K.K, Hamamatsu City 434-8601, Japan

**Keywords:** Breast cancer, Diffuse optical imaging, Total hemoglobin, Glucose metabolism

## Abstract

**Background:**

Near-infrared optical imaging targeting the intrinsic contrast of tissue hemoglobin has emerged as a promising approach for visualization of vascularity in cancer research. We evaluated the usefulness of diffuse optical spectroscopy using time-resolved spectroscopic (TRS) measurements for functional imaging of primary breast cancer.

**Methods:**

Fifty-five consecutive TNM stageI/II patients with histologically proven invasive ductal carcinoma and operable breast tumors (<5 cm) who underwent TRS measurements were enrolled. Thirty (54.5%) patients underwent ^18^F-fluoro-deoxy-glucose (FDG) positron emission tomography with measurement of maximum tumor uptake. TRS was used to obtain oxyhemoglobin, deoxyhemoglobin, and total hemoglobin (tHb) levels from the lesions, surrounding normal tissue, and contralateral normal tissue. Lesions with tHb levels 20% higher than those present in normal tissue were defined as “hotspots,” while others were considered “uniform.” The findings in either tumor type were compared with clinicopathological factors.

**Results:**

“Hotspot” tumors were significantly larger (*P* = 0.002) and exhibited significantly more advanced TNM stage (*P* = 0.01), higher mitotic counts (*P* = 0.01) and higher levels of FDG uptake (*P* = 0.0004) compared with “uniform” tumors; however, other pathological variables were not significantly different between the two groups.

**Conclusions:**

Optical imaging for determination of tHb levels allowed for measurement of tumor vascularity as a function of proliferation and glucose metabolism, which may be useful for prediction of patient prognosis and potential response to treatment.

## Background

Tumor angiogenesis is a vital process in the early phases of cancer progression [1-3]. Of late, functional imaging using near-infrared (NIR) diffuse optical spectroscopy (DOS) has been used to develop noninvasive measurements for detection of primary breast cancer [4-6]. NIR time-resolved DOS (NIR–TRS) systems are portable, have high data acquisition rates, and can detect variations in photon transit times resulting from varying levels of oxyhemoglobin (O_2_Hb) and deoxyhemoglobin (HHb), which characterize optical properties of the tissue in terms of absorption coefficient (*μ*_a_) and decreased scattering coefficient (*μ*_s’_) [7]. Quantification of O_2_Hb and HHb levels in breast tissue allows for the measurement of total hemoglobin (tHb) levels (tHb = O_2_Hb + HHb). Blood volume is directly related to tHb levels, and abnormal tumor vascularization is believed to contribute to local elevation in tHb levels [8]. Optical imaging provides excellent contrast of tHb levels in malignant tumor tissue and surrounding normal tissues, and it has been considered useful for detecting tumor vascularity and differentiating tumors from neighboring tissues [9].

Zhu et al. first reported that an ultrasonography (US)-guided optical imaging device could be used to distinguish early-stage breast cancer from benign lesions and that the lesions showed two-fold higher tHb levels than those observed in benign lesions [10,11]. On the other hand, in a study of 276 patients in a readers-blinded comparison study, Collettini et al., a radiologists’ study group in Germany, reported no significant improvement in diagnostic performance of initial NIR optical tomography for the detection of primary breast cancer compared with the performance of a combination of mammography and optical tomography. This was because of the low spatial resolution of optical imaging [12].

Although the sensitivity for optical detection of tumoral lesions cannot be expected to be excellent, intrinsic optical contrast of malignant tumors, especially local elevation of tHb levels, should correlate with biological and physiological features. We hypothesized that optically visible tumors with locally elevated tHb levels relative to those in the surrounding normal breast tissue have increased angiogenesis and that optically low-contrast tumors are less aggressive. In this study, we prospectively enrolled consecutive, operable, TNM stageI/II patients with relatively small tumors (<5 cm) that were initially diagnosed as invasive ductal carcinoma (IDC) using biopsy. We investigated the potential clinical application of optical imaging as a means of differentiating the unique features of breast cancer.

## Methods

### Patients

We enrolled 88 patients from July 2012 to December 2012 at the Department of Breast Oncology, International Medical Center, Saitama Medical School (Saitama, Japan). Seventy women were diagnosed with IDC using vacuum-assisted biopsy (Mammotome^®^, Johnson & Johnson, USA) after identification of tumors by X-ray mammography, ultrasonography (US), and/or dynamic magnetic resonance imaging (MRI). Specialized breast radiologists used US and/or MRI to determine the clinical size of the lesions. Histopathological analysis of breast cancer, including determination of grade and intrinsic subtype, was performed by at least two experienced pathologists who used hematoxylin–eosin-stained and immunohistochemical-stained slides of all core biopsy and surgical specimens. Two patients with bilateral lesions, eight with large lesions (diameter ≥5 cm), 10 who had already received neoadjuvant therapy, and 13 who were diagnosed with non-IDC or special types of breast carcinoma were excluded. The final study group comprised 55 consecutive TNM stageI/II breast cancer women (62.5%) with IDC (diameter <5 cm) who ranged in age from 22 to 81 years (mean, 58.6 years). The study protocol was approved by the Institutional Review Board of Saitama International Medical Center (Saitama, Japan). Informed consent was obtained from all patients prior to the study.

### TRS breast imaging system

A dual-channel TRS system (TRS20, Hamamatsu Photonics K.K., Japan) was used to measure the optical properties of breast tissue at three wavelengths (760 nm, 800 nm, 834 nm). This system uses a time-correlated single-photon counting (TCSPC) method for measuring temporal response profiles of tissue against optical pulse inputs and enables quantitative analysis of light absorption and scattering in tissue as per the Photon Diffusion Theory [13]. The nonlinear least squares method was used to fit the solution of the photon diffusion equation in the reflectance mode to the observed temporal profiles. The coefficients *μ*_a_ and *μ*_s’_ were obtained at three wavelengths, and the O_2_Hb and HHb levels were calculated from the spectroscopic O_2_Hb and HHb data [14]. Then, the tHb levels were calculated by adding the O_2_Hb and HHb levels.

**Figure 1:**
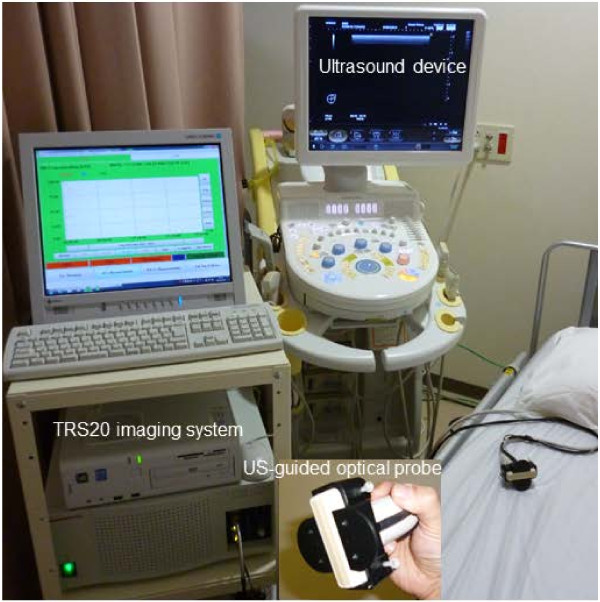
**A dual-channel TRS system.** The patient lies in the supine position on the bed. A US-guided optical probe from the TRS imaging system (TRS20, Hamamatsu Photonics K.K., Japan) is used to acquire measurements of a patient’s breast and define an ROI in which the breast lesion can be measured.

**Figure 2:**
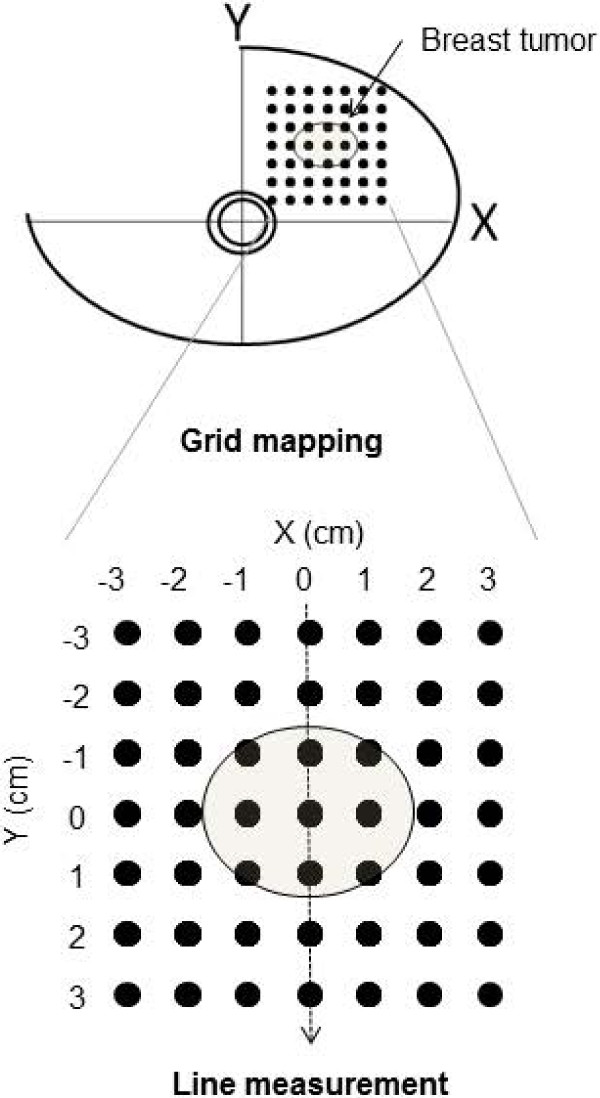
**TRS measurement procedure and 2D hemoglobin map construction.** Optical measurements comprising a grid map over tumor and normal breast tissue are obtained using a handheld probe. The tumor is always located in the center of a map.

The TRS imaging system is presented in Figure 1. A handheld probe with a 3-cm source–detector distance was used to measure the breasts with the patients in a supine position. On the basis of the information obtained from the US system (HI VISION Preirus™, Hitachi, Japan) in which the probe was combined with an optical probe as shown in Figure 1, a 10-mm square grid map (Figure 2) was constructed on the lesion and surrounding normal tissue. The points of maximum tumor size were arrayed in the center of the map. The grid map of a tumor-burdened breast basically comprised 7 × 7 points with a 10-mm interval between two points in the x–y dimension. A minimum of 49 measurement points was obtained for each breast map. Because the spatial resolution of diffused light is poorer than that of US, a lesion region of interest (ROI) used for two-dimensional (2D) image reconstruction of tHb distribution that was at least two-fold larger than that observed by using US in the x–y dimension was chosen. For the contralateral normal breast, a grid map comprising 5 × 5 points with a total of 25 points in the x–y dimension was constructed in the quadrant region corresponding to the lesion. For spline interpolation, 2D image processing, and analysis, custom software (DataGridViewer, version 12; SincereTechnology Corp., Kanagawa, Japan) was used.

**Figure 3:**
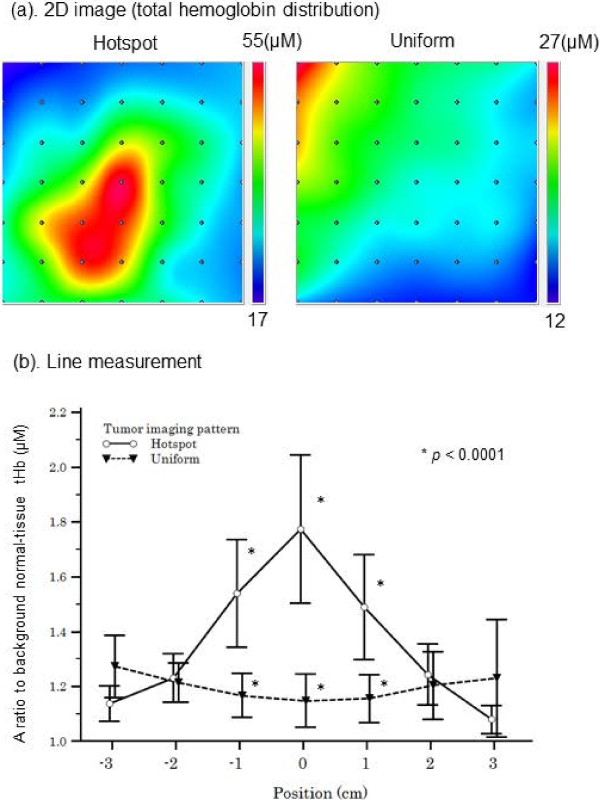
**Optical imaging of tHb level in the breast. (a)** A 2D image of the breast total hemoglobin level (tHb) is constructed by applying a spline interpolation algorithm to the raw data. In this example, maps of “hotspot” and “uniform” patterns are shown. **(b)** A line scan shows that compared with tumors with a “uniform” pattern, tumors with a “hotspot” pattern exhibit a significantly higher ratio of tHb levels to that in the background normal breast tissue.

Average lesion tHb levels were calculated from tissue O_2_Hb and HHb levels obtained using TRS measurement of breast tissue corresponding to the ROI. The measurement procedure and grid maps of tHb levels are shown in Figure 3.

### Nuclear grading system

The nuclear grade of IDC was determined by at least two pathologists according to General Rules for Clinical and Pathological Recording of Breast Cancer, 15^th^ edition [15]. Nuclear atypia and mitotic count scores were classified as low (1) and high (2 and 3).

### Immunohistochemistry

The expression of estrogen receptor (ER), progesterone receptor (PgR), and human epidermal growth factor receptor-2 (HER2) were immunohistochemically examined as a routine for all specimens. Monoclonal anti-ER antibody (clone ID5) (1:100), monoclonal anti-PgR antibody (clone PgR636) (1:100), and the Herceptest kit for HER2 were purchased from Dako (Grostrup, Denmark) and used for immunohistochemical analysis. The method used for immunohistochemistry was as described previously [16]. In brief, the 4 μm-thick sections were deparaffinized in xylene, and dehydrated in a graded ethanol series. Antigen retrieval was carried out by incubation of the tissue sections in a microwave oven in 10 mM sodium citrate (pH 6.0) with 0.1% Tween40 at 120°C for 45 min. After antigen retrieval, the tissue sections were incubated in 0.3% hydrogen peroxide in methanol for 30 min, reacted with the primary antibody for 1–3 h, incubated with dextran polymer reagent conjugated with peroxidase and secondary antibody (envision; Dako, Glostrup, Denmark) for 1 h, and subsequently reacted with 3,3-diaminobenzidine tetrahydrochloride-hydrogen peroxide as the chromogen.

In the present study, a hormone receptor status score of 3+ (≥10% nuclear staining) was regarded as positive while a score of 2+/1+/0 (<10%) was regarded as negative [17]. With regard to HER2 expression, cases with a score of 3+ were judged as showing overexpression. If a score was 2+, fluorescent in situ hybridization (FISH) was performed. When amplification of the HER2 gene using FISH was observed, it was considered to be a positive result [18]. Others were considered to be negative.

### ^18^F-fluoro-deoxy-glucose-PET/CT

Thirty enrolled patients (54.5% total) agreed to undergo ^18^F-fluoro-deoxy-glucose (FDG)^**-**^positron emission tomography (PET)/computed tomography (CT) scans (Biograph-16, Siemens–Asahi Medical Technologies, Tokyo, Japan) at the Department of Nuclear Medicine of our institution. Details of the measurement procedure are as previously described [19,20]. Patients fasted for at least 6 h before the ^18^F-FDG PET/CT study. One hour after intravenous administration of 3.7 Mbq/kg ^18^F-FDG, a transmission scan using CT for attenuation correction and anatomical imaging was acquired for 90 s. PET data were reconstructed via a combination of Fourier rebinning and the ordered subsets expectation maximization at iteration number 3 and subset 8 with attenuation correction based on CT data. An ROI was placed on the primary lesion, including the highest uptake area (circle ROI, diameter 1 cm), and the maximum standardized uptake value (SUV_max_) in the ROI was calculated. SUV was calculated according to the following formula: SUV = ROI activity (MBq/ml)/injected dose (MBq/kg of body weight).

### Binary classification of spatial distribution patterns of lesion tHb

Unique features of tumoral lesions were determined from an evaluation of tissue tHb distribution patterns in the breast map. Spatial variations in the lesion tHb map allowed us to easily locate the maximum optical contrast corresponding to the tumor site. Figure 3(a) shows representative 2D images of tHb distribution patterns in breasts with tumors. We found two qualitative features that enabled differentiation of optically and visually detectable tumors from undetectable ones on the basis of the distribution pattern of tHb. In this study, approximately half the tumors showed excellent tHb contrast against the surrounding normal breast tissue. Others showed equivocal results because of poor contrast between the tumor and surrounding normal tissue. Considering the results presented in Table 1 and from visual assessment, we defined a visually detectable tumor with at least ≥20% local elevation in tHb levels compared with those in both the contralateral breast tissue and surrounding normal tissue as a “hotspot” tumor. The others were described as “uniform” tumors, which did not form a hotspot in the lesion and exhibited a more uniform distribution pattern of tHb or a <20% increase in tissue tHb levels compared with those in the surrounding normal tissue and/or contralateral breast tissue.

**Table 1:** Comparison of hemoglobin parameters of lesion, surrounding tissue, and contralateral tissue

**Optical parameters mean (μM) ± SD**	**Lesion (n = 55)**	**Surrounding tissue (n = 55)**	**Contralateral tissue (n = 55)**	***p *** value^*^
**O**_**2**_**Hb**	**20.4 ± 10.9**	**14.8 ± 9.2**	**15.3 ± 12.8**	**Lesion vs. surrounding tissue:**
				***p*** **= 0.001**
				**Lesion vs. contralateral tissue:**
				***p*** **= 0.03**
**HHb**	**8.9 ± 4**	**6.7 ± 2.8**	**6.8 ± 4.6**	**Lesion vs. surrounding tissue:**
				***p*** **= 0.001**
				**Lesion vs. contralateral tissue:**
				***p*** **= 0.01**
**tHb**	**29.3 ± 14.5**	**21.2 ± 11.7**	**22.1 ± 17.2**	**Lesion vs. surrounding tissue:**
				***p*** **= 0.001**
				**Lesion vs. contralateral tissue:**
				***p*** **= 0.02**

SD, standard deviation; ^*^Student's *t* test.

Figure 3(b) shows the result of TRS line measurement of the breast through the tumor center. Ratios of the tumor tHb and background normal tissue tHb (relative tHb level) were compared between the two groups. The line scan “hotspot” tumors showed a clear maximum on the lesion.

### Statistical analysis

Student’s *t* test was used to calculate significance for comparison between continuous variables because the data followed a normal distribution. The Fisher’s exact test and Pearson’s chi-square test were used to test the statistical significance of the relationship between the independent groups. The Pearson’s correlation coefficient was used to analyze the degree of association between two continuous variables. A level of *P* < 0.05 was considered to indicate statistical significance. Logistic regression analysis was performed to find the best-fitting model to describe the relationship between dichotomous characteristics of tumor tHb distribution (“hotspot” and “uniform” patterns) and a set of the possible discriminators of clinicopathological factors. Statistical software (MedCalc Software, Broekstraat, Belgium) was used for calculation.

## Results

### Baseline characteristics of the patients

Measurement data from a total of 55 tumors were evaluated in this study. There was a minimum 14-day interval (average, 29.5 days; range, 15–55 days) between the diagnostic core needle biopsy and baseline TRS measurements before surgery. Clinicopathological data were obtained from medical records and pathological reports of the surgical specimens. For nine patients (16.4%) who received neoadjuvant endocrine treatment after undergoing TRS scans, pathological data regarding the diagnostic core needle biopsy specimens were obtained for this study. Table 2 presents the patient and tumor characteristics.

**Table 2:** Clinicopathological factor and biomarker results in hotspot versus uniform-patterned breast cancers assigned by tHb optical imaging

**Variables**	**Values**	**Total**	**Hotspot**	**Uniform**	***p *** **value**
**Age**	**Mean(year) ± SD**	**58.6 ± 12.3**	**59.9 ± 11.7**	**56.5 ± 13**	NS^*^
**TNM Stage**	**I**	**25**	**9**	**16**	***p*** **= 0.01**^**†**^
	**II**	**30**	**22**	**8**	
**Tumor size**	**Mean(mm) ± SD**	**21.5 ± 9**	**24.8 ± 9.9**	**17.4 ± 5.6**	***p*** = 0.002^*^
**Nuclear atypia**	**High**	**37**	**20**	**17**	NS^**^
	**Low**	**5**	**3**	**2**	
**Mitosis**	**High**	**14**	**12**	**2**	***p*** = 0.01^**^
	**Low**	**28**	**11**	**17**	
**Nodal status**	**Positive**	**10**	**4**	**6**	NS^**^
	**Negative**	**33**	**20**	**13**	
**ER**	**Positive**	**45**	**24**	**21**	NS^**^
	**Negative**	**6**	**5**	**1**	
**PgR**	**Positive**	**41**	**21**	**20**	NS^**^
	**Negative**	**10**	**8**	**2**	
**HER2**	**Positive**	**6**	**4**	**2**	NS^**^
	**Negative**	**44**	**24**	**20**	
**FDG SUVmax**	**Mean ± SD**	**5 ± 3.3**	**6.6 ± 3.2**	**2.6 ± 1.7**	***p*** = 0.0004^*^

SD, standard deviation; NS, not statistically significant; ^*^Student’s *t* test; ^**^Fisher’s exact test, ^†^Pearson’s chi square.

### Comparison of Hb levels in tumors and normal tissue

Absolute values of tHb, O_2_Hb, and HHb levels were compared between the lesions and the surrounding normal tissue and between the lesions and the normal contralateral breast tissue (Table 1). The mean tHb levels of lesions were 27.6% higher than those in the surrounding normal tissue and 24.6% higher than those in the contralateral tissue.

According to our tHb distribution pattern criteria, 31 tumors (56.4%) were “hotspot” tumors and 24 (43.6%) were “uniform” tumors. There were no significant differences between the two groups in absolute values of O_2_Hb (*P* = 0.9), HHb (*P* = 0.7), or tHb (*P* = 0.8).

### Comparison of clinicopathological factors between “hotspot” and “uniform” tumors

Table 2 shows the patient age, TNM stage, tumor size, nuclear atypia score, mitotic counts, nodal status, ER staining, PgR staining, and HER2 status for the “hotspot” and “uniform” tumors. The “hotspot” tumors showed significantly more advanced stage than “uniform” tumors (*P* = 0.01). The diameters of the “hotspot” tumors were significantly higher than those of the “uniform” tumors (*P* = 0.002). There were no significant differences between the two groups in any other clinicopathological factors: age (*P* = 0.3), nuclear atypia score (*P* = 0.8), nodal stage (*P* = 0.4), ER staining (*P* = 0.3), PgR staining (*P* = 0.2), or HER2 status (*P* = 0.9). The number of “hotspot” tumors showing high mitotic count scores (52.2%) was significantly higher than that of “uniform” tumors showing high scores (10.5%; *P* = 0.01). Tumor SUV measured by FDG PET/CT was significantly higher in “hotspot” tumors than in “uniform” tumors (*P* = 0.0004).

### The relationship between FDG SUV_max_ and tHb

**Figure 4:**
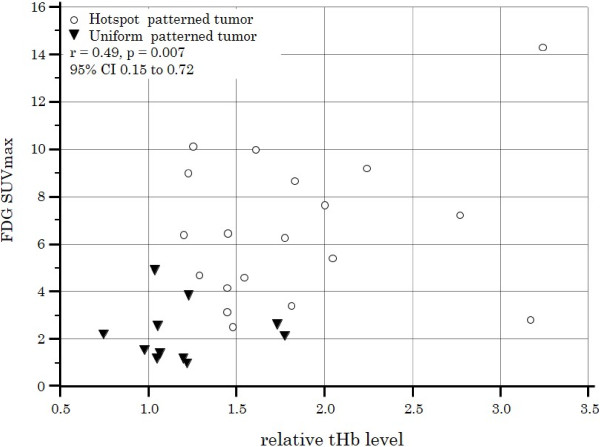
**Correlation between FDG uptake and tumor tHb patterns.** A scatter diagram of two patient groups (“hotspot” and “uniform”) showing a significant relationship between FDG SUV_max_ and relative tHb level (coefficient r = 0.49; 95%CI, 0.15–0.72, *P* = 0.007).

When tumor size, TNM stage, mitotic count, and FDG SUVmax were loaded in logistic regression analysis, none of these variables contributed significantly to the prediction of “hotspot” tumor. Figure 4 shows FDG SUVmax was significantly correlated with relative tHb level of tumor (coefficient r = 0.49; 95% CI, 0.15-0.75, *P* = 0.007).

## Discussion

In this study, we investigated the clinical application of functional NIR–DOS imaging for the measurement of intrinsic contrast of early-stage breast cancer. Significantly higher tHb levels were observed in early-stage breast cancer tumors than in the surrounding normal breast tissue and contralateral normal breast tissue. However, there were a wide range of tHb levels between the individual tumors and the normal tissues, and >40% tumors did not show a clear elevation in tumor tHb levels because of the presence of equal tHb levels in the normal tissue. This finding is understandable because mammary glandular tissue has much denser vascularity compared with fatty tissue, and the absolute values of tissue tHb levels vary among individuals. Tumors progress with the growth of new vessels from pre-existing vessels so that lesion tHb levels continue to correlate with the extent of vascularity in the background normal tissue. In addition, tumor Hb levels are reportedly more sensitive to hormonal fluctuations induced by the menstrual cycle compared with those in the normal breast tissue, with 10%–14% deviation [21]. Therefore, we focused on increased ratios of tHb levels in lesions to tHb levels in the surrounding normal tissue and eventually established a certain criteria for “hotspot” tumors, which were detectable with a ≥20% local elevation in tHb levels relative to that in the normal tissue. The other tumors, which were visually equivocal or not clearly detected by optical imaging, were classified as “uniform” tumors.

The “hotspot” pattern of tHb level was detected in 56% patients with early-stage breast cancer. Tumor size was significantly greater in “hotspot” tumors than in “uniform” ones, but this finding did not act as a predictor of excellent optical contrast because of a remarkable overlap between the two groups. This indicated that the size of a tumor did not dictate its clarity on optical imaging.

Mitotic count score, evaluated as a proliferative marker, was significantly higher in “hotspot” tumors (52.2%) than in “uniform” ones (10.5%; *P* = 0.01). In addition, tumor SUV_max_ measured by FDG PET/CT was a good index for discriminating between “hotspot” and “uniform” tumors. Therefore, high-metabolic tumors should be identifiable by optical imaging because of progressive angiogenesis, but some tumors with low metabolic activity may absorb the NIR light for optical measurements to a lesser degree than that absorbed by the surrounding normal tissue because of less blood retention due to less aggressive neoangiogenesis. A biomarker study conducted by Groves et al. revealed that tumor FDG uptake was significantly associated with angiogenesis as measured by an immunohistochemical bioassay of CD105 for new vessel formation in patients with early-stage breast cancer [22]. This finding suggests that tumor vascularity is closely associated with tumor glycolytic activity.

Cancer cells respond autonomously to hypoxia, switch oxidative phosphorylation in mitochondria to glycolysis, and positively amplify neoangiogenesis [3,23]. Paradoxically, the phenomenon by which these tumors acquire an increased glycolytic rate despite normal tissue oxygen tension is called the Warburg effect [24,25]. Recent research revealed that autonomous upregulation of several oncogenic signaling mechanisms independent of hypoxia, including a PI3K–AKT pathway, transcriptional activity of HIF1, and aberrant function of p53, affects overexpression of glucose transporters and related enzymes. The activation of these mechanisms contributes to hypermetabolism and neoangiogenesis of the tumor [22,26]. Therefore, it is evident that increased glucose metabolism and angiogenesis may be, to some extent, different phenotypical expressions of common underlying genetic and/or physiological processes [27].

Currently, FDG PET/CT attracts the attention of oncologists because the biological basis of FDG uptake in cancer metabolism could be the Warburg effect [28]. The result that elevation of tumor tHb levels relative to those in background normal breast tissue was correlated with high FDG uptake is consistent with the observation of recent research that showed the coupling of increased glucose metabolism of cancer cells to neoangiogenesis and hypoxia. Therefore, these features of “hotspot” and “uniform” patterns can add functional information regarding the physiology of the tumor. For example, early-stage breast cancer patients with “hotspot” tumors could initially be considered chemotherapy candidates in terms of cancer cell activity.

Furthermore, breast cancer is known to have heterogeneous characteristics of gene expression patterns that are strongly associated with prognosis and response to therapy [29]. In the future, we believe that breast cancer may be further classified into types on the basis of spectral differences.

The strength of this study was that we enrolled a homogeneous group of consecutive TNM stageI/II patients with small-size (mean, 21.5 mm) IDC tumors, whereas previous studies on optical breast imaging have included advanced-stage or various histological types of breast cancers [9,11,30].

Functional imaging using DOS has limitations with regard to the identification of tumor location because intense light scattering in tissues leads to low spatial resolution and in-depth information of tissue absorption cannot be assessed [31]. The current study used data from a small patient population. A large prospective study is required to further validate the results.

## Conclusions

Optical imaging of breast cancer tHb levels can potentially contribute to the identification of unique functional features of tumor vascularity that add diagnostic value to cancer management and may assist in the development of a monitoring tool for treatment.

## Abbreviations

DOS: Diffuse optical spectroscopy; TRS: Time-resolved spectroscopy; FDG: ^18^F-fluoro-deoxy-glucose; PET: Positron emission tomography; O2Hb: oxyhemoglobin; HHb: Deoxyhemoglobin; tHb: Total hemoglobin; NIR: Near-infrared; μa: absorption coefficient; μs: Reduced scattering coefficient; US: Ultrasonography; IDC: Invasive ductal carcinoma; TCSPC: Time-correlated single-photon counting; ROI: Region of interest; 2D: Two-dimensional; FISH: Fluorescent in situ hybridization; SUV: Standardized uptake value; ER: Estrogen receptor; PgR: Progesterone receptor; HER2: Human epidermal growth factor receptor-2.

## Competing interests

SU, NN, KM, TS, HS, EH, HS, OS, IK, AO, and TS had no competing interests. HS, MO, and YY are employees of Hamamatsu Photonics K.K. They have not applied for any patents related to this study.

## Authors’ contributions

SU conceived and designed the study, conducted measurements, analyzed the data, and performed the statistical and graphical analysis. NN conducted measurements and analyzed the data with SU. SU and NN acquired funding in the form of a Hidaka research grant from Saitama Medical University (SMU). KM, TS, HS, EH, HS, and AO registered patients eligible for the study. HS and MO advised us on technical issues and maintained the TRS imaging system. IK participated in FDG PET image acquisition. TS was a significant contributor to the study design, manuscript content, and organization. All authors read and approved the final manuscript.

## Authors’ information

Shigeto Ueda, MD is a breast surgeon and completed his PhD at SMU. Research interests include functional PET imaging and diffuse optical spectroscopy. He currently works as an assistant professor at SMU. Noriko Nakamiya, MD is a breast surgeon in the Department of Breast Oncology at SMU. Her research interests are in early breast cancer detection using mammography and optical spectroscopy. Kazuo Matsuura, MD, PhD is an associate professor at SMU. He is a breast surgeon. His research interests include molecular biology and cancer immunology.

Takashi Shigekawa, MD, PhD is an assistant professor in SMU. He is a breast surgeon. Hiroshi Sano, MD, PhD is an assistant professor at SMU. He is a breast surgeon. Eiko Hirokawa, MD is an assistant professor at SMU. She is a breast plastic surgeon. Hiroko Shimada, MD is an assistant professor at SMU. She is a breast surgeon. Hiroaki Suzuki, PhD is a researcher at Hamamatsu Photonics K.K. He maintained the TRS imaging system. Motoki Oda, PhD is a researcher at Hamamatsu Photonics K.K. He also developed and improved the TRS imaging system. Yutaka Yamashita, PhD is the chief researcher in the Central Research Laboratory of Hamamatsu Photonics K.K. He developed the TRS imaging system. Ichiei Kuji, MD, PhD is a radiologist and a professor in the Department of Nuclear Medicine at SMU. His research interests include cancer imaging using functional PET. He aids in the detection and diagnosis of breast cancer using FDG PET scans. Akihiko Osaki, MD, PhD is a breast surgeon and a professor in the Department of Breast Oncology at SMU. His research interests include early detection of breast cancer using mammography and optical spectroscopy. Toshiaki Saeki is a vice president at SMU and the chief professor in the Department of Breast Oncology. His research interests include design of clinical trials, molecular biology of cancer, cancer imaging, and development of molecular targeting agents. His interests in the field of biophotonics are centered on research and technology development of diffuse optical imaging for applications in breast cancer research.

## Pre-publication history

The pre-publication history for this paper can be accessed here:

http://www.biomedcentral.com/1471-2407/13/514/prepub
